# Platelet Metabolic Flexibility: A Matter of Substrate and Location

**DOI:** 10.3390/cells12131802

**Published:** 2023-07-07

**Authors:** Silvia Ravera, Maria Grazia Signorello, Isabella Panfoli

**Affiliations:** 1 Department of Experimental Medicine, University of Genoa, 16132 Genoa, Italy; silvia.ravera@unige.it; 2 Department of Pharmacy (DIFAR), University of Genoa, 16132 Genoa, Italy; mariagrazia.signorello@unige.it

**Keywords:** bioenergetics, metabolism, oxidative phosphorylation, platelet

## Abstract

Platelets are cellular elements that are physiologically involved in hemostasis, inflammation, thrombotic events, and various human diseases. There is a link between the activation of platelets and their metabolism. Platelets possess considerable metabolic versatility. Although the role of platelets in hemostasis and inflammation is known, our current understanding of platelet metabolism in terms of substrate preference is limited. Platelet activation triggers an oxidative metabolism increase to sustain energy requirements better than aerobic glycolysis alone. In addition, platelets possess extra-mitochondrial oxidative phosphorylation, which could be one of the sources of chemical energy required for platelet activation. This review aims to provide an overview of flexible platelet metabolism, focusing on the role of metabolic compartmentalization in substrate preference, since the metabolic flexibility of stimulated platelets could depend on subcellular localization and functional timing. Thus, developing a detailed understanding of the link between platelet activation and metabolic changes is crucial for improving human health.

## 1. Introduction

Platelets are small anucleate cells derived from the cytoplasmic fragmentation of megakaryocytes [1]. Platelets have been incorrectly considered simple cells, whereas they display rather complex signal transduction machinery typical of excitable cells, and metabolic capacity. Platelets are often taken as a model for neurochemical, biochemical, and molecular biology studies. Although platelets were the last circulating cell to be identified [2], their central role in hemostasis is well established today [3]. Platelets help to form a thrombus without occluding the vessel. In primary hemostasis, platelets adhere to the damaged endothelial wall, through a process requiring multiple signaling cascades highly that are dependent on glycoproteins (GPs) expressed on the platelet surface [4]. In detail, platelet glycoprotein IIb/IIIa (GPIIb/IIIa) receptor (integrin αIIbβ3) mediates platelet aggregation, undergoing a conformational change to a high-affinity ligand-binding state.

### 1.1. Platelets in Diseases

Platelets play a pivotal role in myocardial infarction, stroke, vascular disease [5], atherothrombosis [6], metabolic syndrome [7], and cancer [8]. Metabolic syndrome (MS) pathophysiology includes insulin resistance, oxidative stress, and endothelial dysfunction and is associated with platelet hyperactivity [9]. In Chinese women, the mean platelet volume (MPV) has been found to increase and has been inversely associated with the risk of developing MS [7]. Also, the platelet count and distribution extent increased in subjects with MS [10]. Platelets promote tumor progression and metastasis. The underlying mechanism includes direct interactions of tumor cells with platelets, which are activated and promote increased thromboembolic complications [8]. In addition, platelets act in the tumor microenvironment by releasing various chemokines and controlling angiogenesis via the release of α-granules containing pro-angiogenic factors [11]. Platelets contribute to the dissemination of cancer cells in the circulation, adhering to them, and thereby supporting their metastatic potential and tumor survival [8].

Platelets have been described as “inflammatory cells” as they contribute to the pathogenesis of inflammatory diseases. Depending on the context, platelet immune functions can be beneficial [12]. Platelets are also involved in inflammatory and allergic reactions [13], in some gastroenterological [14,15,16], renal [17], and dermatological disorders [18], as well as in viral [19,20], bacterial [20], and parasitic [21] diseases. Platelets play a central role in primary hemostatic defense by adhering to damaged vessels and becoming activated [22]. Platelet activation is conducted by signaling pathways after receptor–ligand interactions [23]. In detail, following vascular damage, the receptor GPIbα, following activation, binds to P-selectin (CD62P) and vWF [24]. A complex of signaling pathways, culminating in increased intracellular Ca^2+^ concentration and the subsequent production of lipid mediators, causes platelet cytoskeleton reorganization, shape changes, granule secretion, and aggregation [25]. Platelets play a pivotal role in arterial thrombosis [26]. In fact, crosstalk exists between inflammation and thrombosis in that the former can cause thrombosis, which, in turn, may exacerbate inflammation [4], a phenomenon called thrombo-inflammation [27]. Thrombosis can be triggered by interactions mediated by platelet CD62P binding to its receptor (PSGL-1) on polymorphonuclear leukocytes, leading to neutrophil extracellular trap (NET) formation, which was shown to play a pivotal role in thrombus formation [28]. Moreover, NET formation is frequent in cancer patients and is associated with the incidence of thrombosis and cancer-related coagulopathy [8]. Platelets communicate with inflammatory cells by releasing cytokines and lipid mediators [29]; they also directly interact with circulating neutrophils, monocytes, and T lymphocytes [30]. For example, platelets are critical mediators of thrombo-inflammation during the ischemia–reperfusion (IR) injury response [31]. So-called thrombo-inflammation was also found to be involved in coronavirus disease 2019 (COVID-19) [32]. Platelets from hospitalized COVID-19 patients were fewer and displayed a transcriptomic profile characteristic of hyperreactive and immature platelets, a condition associated with increased critical illness [33]. This condition is probably due to alterations in the platelet transcriptome caused by SARS-CoV-2 virions entering megakaryocytes [33]. 

### 1.2. Platelet Metabolism 

Despite their apparent simplicity, platelets are highly metabolically active cells [34]. There is a consensus on the unique metabolic flexibility of platelets [35,36]. Upon activation, platelets initiate energy-demanding processes [37], switching between substrates and metabolic pathways. However, a remarkable difference in views exists in the literature regarding the metabolic pathways through which platelets gather the chemical energy needed for activation. Some studies report a switch to aerobic glycolysis (the conversion of glucose to lactate in the presence of oxygen) upon platelet activation [35,38,39]. Other studies have considered the switch to oxidative metabolism a fundamental requirement for the transition to an activated state [36,38,40]. Platelets’ metabolic substrate preferences are not yet completely clear. Some authors recently deepened their previous studies, stating that aerobic glycolysis fuels platelet activation and concluding that fatty acid (FA) β-oxidation sustains ATP levels in activated platelets [41]. The study utilized inhibitors of FA oxidation on stimulated platelets, showing that the former significantly impairs oxidative phosphorylation (OxPhos) and ATP levels, inhibiting granule release and thrombus formation, which glycolysis could not compensate [38]. It was observed that glycolysis could not compensate for impaired OxPhos in hypoxic conditions during platelet aggregation [38]. Thrombin addition causes a rapid increase (by about 50%) in platelets’ oxygen consumption, suggesting a contribution of platelet mitochondria to the energy requirement for their aggregation [36]. Most of the oxygen consumed by platelets was reported to be related to ATP production [42]. Resting platelets rely on glycolysis [43], while OxPhos is active in both the resting and activated platelet states, and it utilizes glucose and glycogen, FA, and minimal glutamine [44]. Platelets’ transition to an activated state promotes the rapid uptake of glucose. GLUT3 deletion abolishes agonist-mediated glucose uptake [42,44]. In fact, platelets express glucose transporters 1 (GLUT1) and 3 (GLUT3). Most GLUT3 is present in α-granule membranes (85%) and is translocated to the platelet plasma membrane upon activation [42,44]. GLUT3 plays a pivotal role in platelet activation [45]. Platelets display considerable glycogen turnover. The importance of glycogen depends on platelet activation status and extracellular glucose availability, being more important in hypoglycemic rather than hyperglycemic extracellular conditions [46]. Glycogenolysis is fundamental in blood clot retraction [47]. Platelets give thrombus its physiologic structure, this encompasses compaction of the fibrin network and compression of the embedded erythrocytes in the clot core [48]. Glycogen phosphorylase inhibitors block clot contraction, which can be reversed by alternative metabolic substrates [46]. Some pathological conditions, such as asthma, are associated with abnormal clot architecture and lower clot retraction rates [49]. Fatty acid (FA) β-oxidation supplies most ATP in stimulated platelets during granule secretion and thrombus formation [41] and can compensate for a reduction in glucose availability [40,44]. However, the inhibition of FA oxidation and/or glutaminolysis cannot prevent platelet aggregation [36]. Inhibiting glucose metabolism with 2-deoxy glucose (2-DG) abolishes thrombin-mediated lactate production [36]. On the other hand, double GLUT1/3 knockout platelets are involved in oxidative metabolism, relying on non-glycolytic substrates [44]. Recent data support the idea that platelet activation ensues an increase in both glycolysis and OxPhos to fuel their energy metabolism [38]. Consistently, only the combination of 2-DG and oligomycin inhibits platelet aggregation [35]. The increase in glucose uptake upon platelet activation may be interpreted as demonstrative of a predominant shift to an aerobic glycolytic phenotype, even though a rise in oxygen consumption is also observed [35]. On the other hand, it can be supposed that glucose empowers platelets via both glycolysis and OxPhos. The idea of a double fate for glucose inside the activated platelet also appears to be supported by the data, suggesting the existence of two pools of glucose with different metabolic fates, with that from glycogen being selectively directed to the TCA cycle and oxidation, while the extra-platelet glucose would produce lactate through aerobic glycolysis [46]. The metabolic plasticity of platelets might be taken to a different level, as they contain two compartments (inside and outside the mitochondrion) involved in the use of glucose. Their support of OxPhos implies that the two locations can utilize fatty acids to fuel mitochondria and glucose to fuel ecto-OxPhos [50]. In this respect, our previous data demonstrated the existence of OxPhos capacity outside the mitochondrion in the endoplasmic reticulum (RE) [50]. In addition, circulating blood platelets consumed oxygen and synthesized ATP in the presence of NADH, a substrate not readily permeant into mitochondria and not affected by atractyloside, an inhibitor of the adenine nucleotide translocase [50]. NADH-stimulated OxPhos increased in the presence of thrombin [50]. Consistent with this, a recent study utilizing isotopically nonstationary carbon (13C) metabolic flux analysis in platelets found that at rest, in addition to glucose, platelets preferentially metabolized acetate via the tricarboxylic acid cycle [40]. By contrast, thrombin addition increased their uptake of glucose 3-fold, but not of acetate, and stimulated glucose consumption 4.5-fold but lactate production just 3.7-fold, suggesting an additional metabolic destiny for glucose outside glycolysis [40]. It seems that platelets’ overall metabolic flexibility needs to encompass the fourth dimension, i.e., the timing of the platelets’ physiologic function, in variable and flexible proportions that can change over time to adapt to the muted environmental conditions. Activated platelets undergo exhausting circumstances, involving changes in shape and aggregation status, that ultimately confine them to virtually oxygen- and substrate-free space, i.e., a blood clot [51]. This vision may shed new light on the observation that although mitochondrial platelet content is very low, its basal ATP turnover is higher than that in leukocytes [52]. Circulating platelets are known to contain a negligible number of mitochondria, which are small, and have few cristae [50,53,54]. Stereological measurements demonstrated that human platelets contain very few mitochondria, with some actually being devoid of them [50]. It is tempting to presume that part of activated platelet OxPhos is fueled by the conversion of glycolysis-derived pyruvate in Acetyl CoA by cytosolic pyruvate dehydrogenase. Notably, it was reported that UK5099 (Mitochondrial Pyruvate Carrier Inhibitor) does not inhibit platelet aggregation induced by thrombin [55]. 

### 1.3. Platelets’ Metabolic Plasticity: Adaptation to Energy Demands

The physiological role that activated platelets play places them in diverse conditions in terms of energy demand and substrate availability, and in changing locations over time. Platelets are brought from their quiescent state in the blood flow to an activated state in close connection with the membranes of endothelia and damaged tissues, and eventually to the core of the clot that these are promoting. To adapt to continually changing conditions, their ability to simultaneously exploit double-sided OxPhos, utilizing glucose in the cytosol and FA inside the mitochondrion, can enable platelets to respond to the changing conditions. In conclusion, the bioenergetic profiling of the platelet metabolic microenvironment, including the intra- and extra-mitochondrial compartments of platelets, is desirable to understand how these influence multiple physiological and pathological conditions. A shift toward glycolysis would be a last resort, as suggested by the observation that platelets from pulmonary hypertension patients displayed a metabolic shift towards glycolysis compared with healthy controls [56]. We believe that a better understanding of platelet oxidative metabolism would allow for deeper knowledge of their physiology and the designing of novel treatments targeting the role of platelets in many human diseases. 

### 1.4. Platelets’ Metabolic Plasticity—The Other Side of the Coin: Production of Oxidative Stress

Since, in addition to aerobic glycolysis, activated platelets are fueled by intra- and extra-mitochondrial OxPhos, it is possible to hypothesize that hyperactivation causes excess reactive oxygen species (ROS) production, not only at the level of the mitochondrial matrix but also directly in the cytosol, inducing an oxidative insult. On the other hand, ROS such as O^2−^, OH^−^, ONOO^−^, and H_2_O_2_ are central to cellular redox regulation as signaling molecules, but there can be a switch between the beneficial and deleterious actions of ROS [57,58].

**Figure 1:**
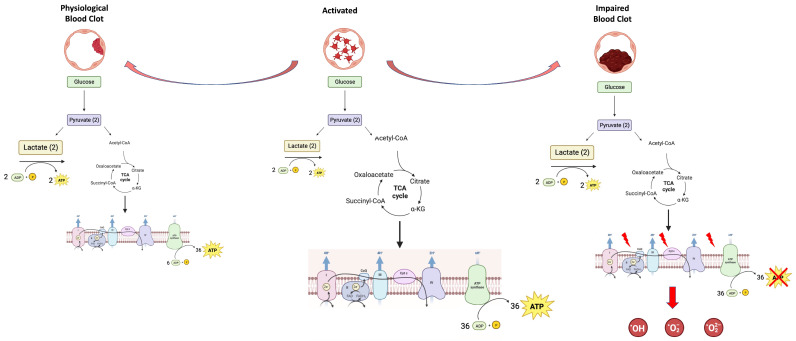
The physiological or pathological formation of the blood clot depends on the platelets’ metabolic plasticity.

Oxidative stress, resulting from an imbalance in ROS production and the antioxidant capacity of a cell, plays a pivotal role in cardiovascular diseases [59]. Risk factors for atherosclerosis, including smoking, diabetes, high blood pressure, and hypercholesterolemia, increase ROS production [60,61,62], ultimately leading to cardiovascular disease [63]. Reactive species such as NO^∙^, ONOO^−^, and HOCl have been reported to impact hemostasis not only via their involvement in atherosclerosis but also via direct action on platelets and/or plasma factors [64,65,66,67]. Mitochondria are the principal ROS producers, especially when uncoupled [68], although the activity of some enzymes, such as superoxide dismutase, nitric oxide synthase, and especially NADPH oxidase (Nox), can generate ROS in platelets [69,70,71], which, in turn, produce significant levels of ROS upon agonist stimulation [72,73]. Increased ROS production and inflammation also activate coagulation [4], which lead to a pro-inflammatory environment, in turn, making them tightly connected to thrombosis. It was shown that, in prothrombotic conditions, i.e., in continuous activation, platelets become refractory, impairing clot contraction and, in turn, producing weaker thrombi that are predisposed to embolization [48] (Figure 1). 

Antiplatelet drugs are a group of chemically diverse medications representing a cornerstone of therapy for atherosclerotic cardiovascular diseases, especially in secondary prevention [74]. These encompass acetylsalicylic acid, whose target is prostaglandin-endoperoxide synthase (PTGS); thienopyridines (clopidogrel and prasugrel) and a cyclopentyltriazolo-pyrimidine (ticagrelor), which inhibit the purinergic P2Y receptor responsible for ADP-induced platelet aggregation; piperidine (tirofiban), an antibody fragment (abciximab); and a cyclic heptapeptide (eptifibatide) preventing GPIIb/IIIa receptor interaction with adhesive ligands. A considerable number of natural drugs (more than 50 different kinds) with inhibitory effects on platelet activation are also known, including flavonoids, saponins, terpenoids, and others [75]. A deeper understanding of the energy metabolism underlying platelet activation and underlying ROS-mediated platelet functional impairment is crucial to the design of an effective anti-thrombotic strategy.

### 1.5. Adenyl Nucleotides beyond Energetics in Platelets

**Figure 2:**
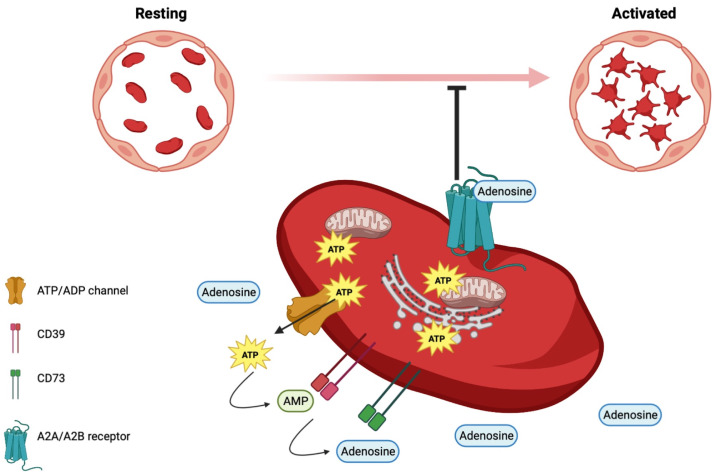
Role of adenosine in platelets.

The antithrombotic properties of intact vascular endothelia, due to the production of prostacyclin, nitric oxide, and adenosine, are lost in the event of endothelial damage. When the endothelium becomes prothrombotic, it promotes platelet activation [76,77]. Activated platelets, in turn, secrete ATP and ADP, building an ecto-ATP/ADP pool, which elicits platelet aggregation, acting on purinergic P2Y1 receptors. In fact, nucleotides are key regulators of platelet activation [78]. An ecto-ATP/ADP pool is built, on which the ectonucleoside triphosphate diphosphohydrolase-1 (CD39), expressed on the vasculature and platelets, hydrolyzes ATP to ADP and AMP. The latter is hydrolyzed to adenosine by ecto-5′-nucleotidase (CD73) [79]. The binding of adenosine to purinergic A2A and A2B receptors on platelets inhibits their activation, thus exerting anti-thrombotic and anti-inflammatory effects [80] (Figure 2). CD39 activity is on the borderline between inflammation and thrombosis [79]. Interestingly, adenosine is a biomarker candidate in the prediction of brain damage in very low-birth-weight premature infants [81].

### 1.6. Omics Approach: A New Strategy for Studying Platelet Biology

Along with biochemical approaches, multi-omics technologies have been employed to investigate platelet biology in health and disease, increasing knowledge of the molecular mechanisms regulating platelet function and crosstalk with other cells. In detail, thanks to ‘omics’ profiling, researchers have gathered new insights into platelet functions. Platelets can bridge basic and clinical studies as they are associated with pathophysiological processes beyond thrombosis, as they control inflammatory, immune, and infection processes and vascular integrity [82].

The first study on the platelet proteome dates back to 1979 and used two-dimensional gel electrophoresis, estimating that platelets contained more than 3000 proteins [83]. By applying new technologies, other recent studies have confirmed these data as the first comprehensive and quantitative proteomic analysis of resting human platelets from healthy subjects has reported the presence of 4000 proteins, providing a first set of data for subsequent studies [84]. Subsequently, on the heels of these proteomic analyses, many studies have addressed both the resting and activated platelet proteome, helping to identify some specific platelet proteome subgroups (reviewed in [85,86]). For example, post-translational modifications of platelet proteins, such as glycosylation or phosphorylation, that are relevant to platelet function in various contexts have also been addressed. In detail, phospho-proteome analyses have revealed phosphorylation patterns in activated platelets, helping to define the molecular mechanisms involved in platelet adhesion, secretion, and aggregation responses [87]. Studies of the glycosylated proteome have shown a principal role in the N-linked glycosylation of adhesive proteins following binding to collagen. Through integrative proteomic and glycoproteomic analysis of collagen−platelet interactive proteins with N-glycan manipulation, it was demonstrated that the interaction of platelet-adhesive receptors with collagen is highly N-glycan-regulated, with glycans on many receptors playing positive roles in collagen binding, and with glycans on other platelet glycoproteins exhibiting inhibitory effects on binding to collagen [88]. The platelet ubiquitinome, another post-translational modification, was analyzed, showing that human platelet proteins undergo extensive protein ubiquitylation during activation [89]. In detail, the study revealed 1634 ubiquitylated peptides derived from 691 proteins, proposing the ubiquitin machinery as a possible target for new therapies [89].

The omics approach has also helped define the role of nucleic acids in platelets. Although, until a few years ago, the RNA contained in platelets was considered vestigial, the analysis of spliceosomes has revealed that mRNA processing and ribosomal mRNA translation occur in platelets, together with protein processing and trafficking, even in the absence of a nucleus [90]. Moreover, other genomic and transcriptomic studies have been conducted to investigate platelet characteristics and residual gene expression [91,92,93], observing that despite the absence of DNA, the platelet transcriptome varies in response to external stimuli using alternative splicing. For example, a whole-genome sequencing study confirmed that the SVEP1 gene is related to coronary artery disease risk [93], establishing a basis for genomic analyses in cases of a family history of cardiovascular risk. Thanks to the integration of different genomic studies on platelets, at first, the Blueprint consortium generated a considerable database cataloging RNAs in blood cell types [94], and, subsequently, a comparative study that combines the human platelet proteome with the platelet and megakaryocyte transcriptomes [94], generating a proteomic map of a human platelet. The classified transcriptome–proteome profile of platelets demonstrated a remarkable quantitative similarity between the platelet and megakaryocyte transcriptomes [92]. Hung et al. classified 5200 platelet-expressed proteins and 14,800 protein-coding transcripts, suggesting that the proteome of platelets may be larger than previously hypothesized [92]. In addition, Simon et al. demonstrated that age and gender differences exist in human platelets in miRNA and mRNA, several of which are targets of the former, in more than seven thousand genes [95]. The authors also developed a specific publicly accessible miRNA-mRNA network (www.plateletomics.com). An example of how the combination of protein and genomic approaches helps when investigating the interaction between platelets and other cells is demonstrated in the manuscript by Mantini et al., in which platelet quantitative transcriptomics and proteomics of pancreatic adenocarcinoma and benign disease were performed, applying, for the first-time, parallel omics approaches using Next-Generation Sequencing (NGS) and LC-MS/MS. Data show that platelets can also influence tumor growth and metastasis in pancreatic ductal adenocarcinoma due to a change in the proteomic profile of platelets. In other words, pancreatic tumor cells are able to “educate” platelets to change their biological repertoire through the dysregulation of miRNAs and splicing factors, to support cancer growth and dissemination [96]. 

Since the wide range of lipids in platelets regulates the main aspects of platelet function [97], mass spectrometry-based high-throughput lipidomic analysis has been applied to identify and quantify platelet lipid species. The first quantitative analysis of the murine platelet lipidome reported that platelets contain almost 400 lipid species [98]. The same authors compared the lipidomic profiles of resting and activated murine platelets, which were validated in human platelets. It was found that about 20% of the platelet lipidome is changed upon activation and that these changes mainly involve phospholipids containing arachidonic acid [99]. Consistent with previous data, a study showed that membrane remodeling is required to support the energetic demand of platelet activation, with a pivotal role of the substrates provided by cytosolic Phospholipase A2 (cPLA2) activity, such as fatty acids that are oxidized by β-oxidation. In fact, it was reported that the platelet lipidome changed during platelet thrombin activation and that this different behavior is inhibited by aspirin [100]. In addition, Slatter et al. also demonstrated that the cPLA2 block causes a slowdown in β-oxidation metabolism, suggesting a link between the inflammation signal and the modulation of energy metabolism [100]. Altered lipidomic profiles have also been observed in patients with coronary artery disease [101], favoring a thrombotic predisposition. In detail, circulating platelets from acute coronary syndrome patients show higher levels of oxidized LDL cholesterol than those in healthy subjects, which alters the platelets’ redox status, influencing their function. On the other hand, Owens et al. have already demonstrated that hyperlipidemic patients display high cholesterol amounts in platelet membranes [102]. Recently, it was demonstrated that the lipidomic signature of platelets from COVID-19 patients is different to that in healthy controls, with increased levels of ganglioside GM3 possibly related to the prothrombotic complications of the disease [103]. Therefore, the results cited above show how the multi-omics approach to analyzing platelets can provide a complete picture of their content and biology, helping to identify new markers that can be employed as prognostic or therapeutic targets.

## 2. Conclusions

Over the years, the metabolism of platelets has been studied by different groups, which have reached different conclusions as to which metabolism most supports the energy demands of platelets. However, these differences are mainly based on the several conditions in which platelets are studied, i.e., resting or activated. Therefore, this review aims to match the different points of view, describing platelets’ metabolic plasticity according to their physiological or pathological conditions. We conclude that platelets are cells with a high degree of flexibility in modulating energy metabolism and the substrates that support it to meet their metabolic demands. However, to fully understand platelet biology, it is necessary to interpenetrate metabolic data with multi-omics analysis results to understand how changes in the genomic (miRNA and mRNA), protein, and lipid heritage of platelets affect their physiological and pathological roles.

## Data Availability

Not applicable.
